# Intra- and interpersonal variation in body surface potentials of healthy subjects

**DOI:** 10.1016/j.hroo.2025.01.016

**Published:** 2025-01-31

**Authors:** Iris van der Schaaf, Manon Kloosterman, Deepthi Priya Chandrasekaran, Peter Loh, Johan de Bie, Peter M. van Dam

**Affiliations:** 1 Department of Cardiology, University Medical Center Utrecht, Utrecht, The Netherlands; 2 Department of Electrical, Electronic, and Information Engineering “Guglielmo Marconi”, Bologna, Italy; 3 ECG Excellence, Nieuwerbrug aan den Rijn, The Netherlands

**Keywords:** Body surface mapping, Electrocardiogram, Healthy subjects, Interpersonal variation, Intrapersonal variation

## Abstract

**Background:**

Body surface potential mapping (BSPM) can provide a detailed assessment of cardiac electrical activity and might be of potential added benefit in multiple cardiac diseases. Normal intra- and interpersonal variation in BSPM is not clearly described and could be of use in the distinction between normal variation and cardiac disease development.

**Objective:**

The purpose of this study was to describe the effects of normal respiration, changes in body position, repeated electrode placement, and heart rate differences on BSPM signals in a healthy population.

**Methods:**

Sixty-seven–lead BSPM was performed in healthy individuals during the resting supine position, a reclined position of 45°, an exercise-increased heart rate, and a follow-up measurement in the resting supine position after 1 week to determine the effect of repeated electrode placement. R-, S- and T-wave amplitudes in all leads were compared between the baseline supine position and the aforementioned conditions.

**Results:**

Ten subjects were included {5 (50%) male; median age 28 years (interquartile range [IQR] 26–30 years)}. The R-wave showed the greatest amplitude variation across all conditions, with the largest changes caused by repeated electrode placement (maximum decrease –0.63 mV [IQR −0.69 to −0.22 mV]) and normal respiration (maximum increase 0.32 mV [IQR 0.08–0.55 mV]) and the smallest changes due to reclined position (maximum decrease −0.23 mV [IQR −0.28 to −0.15 mV]). Electrodes near standard precordial positions were most affected. The exercise-increased heart rate reduced the R-wave amplitude in left-sided electrodes and increased the S-wave amplitude in middle superior electrodes. T-wave amplitude generally increased after exercise.

**Conclusion:**

Normal intrapersonal variation in BSPM signals was analyzed. Repeated electrode placement and normal respiration caused the largest amplitude changes. These findings may help differentiate normal variation from pathological changes in BSPM.

## Graphical abstract

**Figure:**
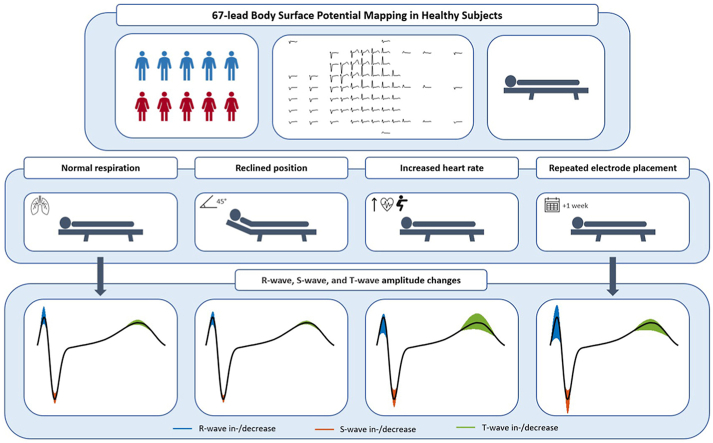

**Key Findings**

▪ Normal respiration and repeated electrode placement caused the most significant changes in R-, S-, and T-wave amplitudes, while small amplitude changes were observed during the reclined position.
▪ R-wave amplitude changes were largest, and T-wave amplitude changes were smallest.
▪ Exercise generally caused an R-wave decrease, a lead-dependent S-wave increase or decrease, and a T-wave increase.
▪ Electrodes closer to the heart were prone to greater amplitude changes than electrodes further from the heart.

## Introduction

Body surface potential mapping (BSPM) is a noninvasive technique that provides a more detailed assessment of cardiac electrical activity than does the standard 12-lead electrocardiogram (ECG). It has been proven useful in multiple cardiac diseases, such as diagnosis of acute myocardial infarction, Brugada syndrome, and assessment of atrial activation in atrial fibrillation.^1^, ^2^, ^3^ Previous research from our group also showed the potential benefit of BSPM in the early detection of disease onset in asymptomatic plakophilin-2-pathogenic variant carriers who are at an increased risk of developing arrhythmogenic cardiomyopathy.^4^ Currently, our group is focused on follow-up of these subjects and performing follow-up BSPM. However, the effects of physiological and external factors such as breathing and repeated placement of electrodes are not as well described in BSPM as they are in the 12-lead ECG.^5^, ^6^, ^7^, ^8^ It is of importance to study these effects, as it may be of aid to distinguish between subtle signs of disease progression and normal intrapersonal variation when comparing 2 consecutive BSPMs. The results of this study are also applicable to 12-lead ECG interpretation, as the standard lead positions are incorporated in BSPM. Therefore, our aim is to study the effects of normal respiration, changes in body position, repeated electrode placement, and differences in heart rate on BSPM signals in a healthy population.

## Methods

### Study population

Subjects were eligible for inclusion if they were ≥18 years, had a normal 12-lead ECG with sinus rhythm, and had no history of cardiac complaints (shortness of breath, palpitations, or chest pain). The study was performed according to the Declaration of Helsinki, and the study protocols were approved by the medical ethics review board of the University Medical Center Utrecht (#17-907). All subjects provided oral and written informed consent before inclusion.

### Data collection

**Figure 1:**
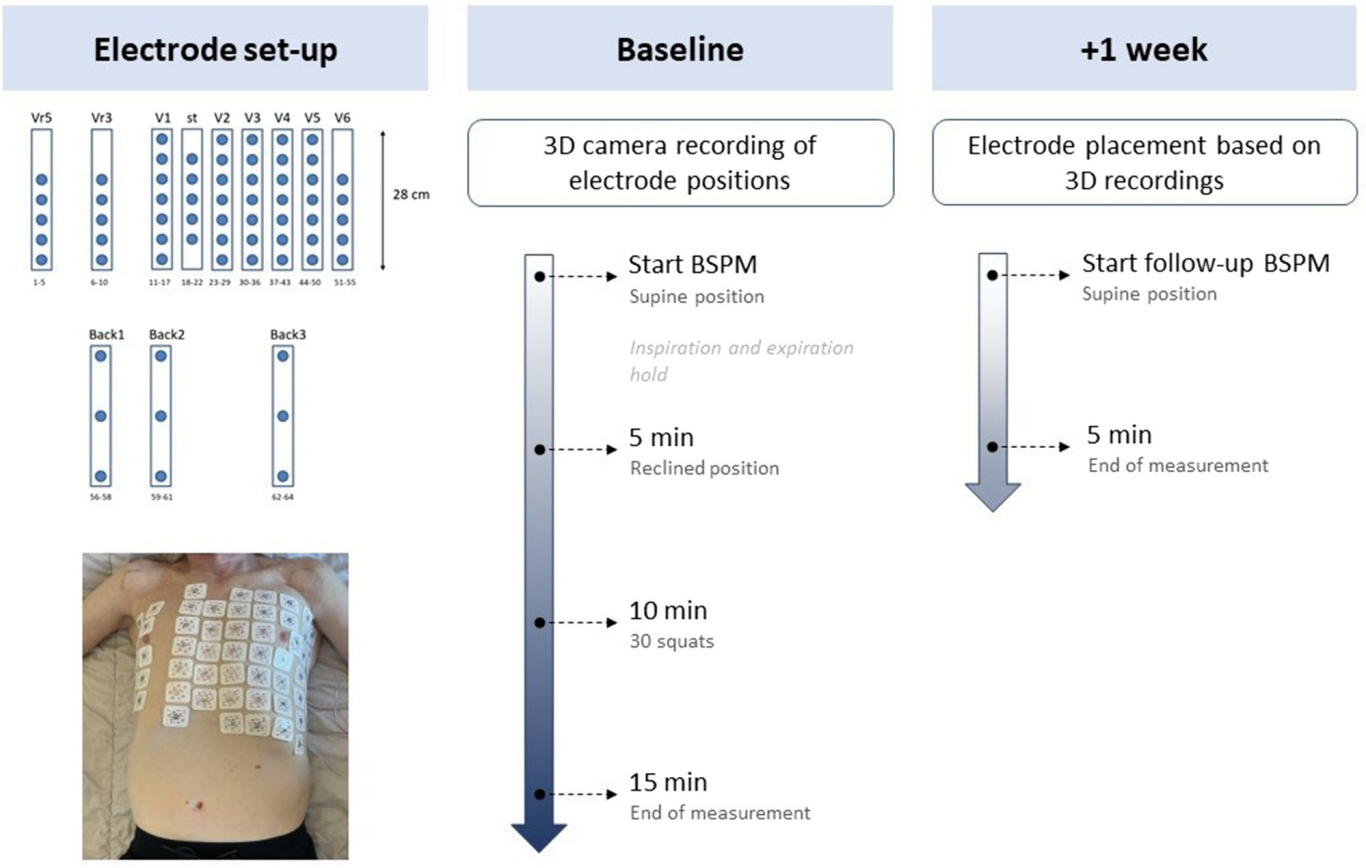
Setup of the torso electrodes from the 67-lead body surface mapping (BSPM) system, and the study workflow showing the procedures performed at baseline and a week later during follow-up BSPM. 3D = 3-dimensional.

The workflow of the study is displayed in Figure 1. Sixty-seven–lead BSPM was performed (BioSemi, Amsterdam, The Netherlands), which included 9 electrodes on the back, 55 chest electrodes, and 3 extremity electrodes positioned in the standard configuration. Wilson’s Central Terminal was derived from the extremity electrodes and used as a reference for the chest and back electrodes. The position between the electrodes was standardized using 12 vertical strips, with 4 cm between the chest electrodes and 9 cm between the back electrodes. The horizontal position of the strips was standardized by positioning the third electrode of the electrode strip on the sternum (electrode 20) at the location of the xyphoid process. The position of the electrodes was recorded with a 3-dimensional (3D) camera (Intel RealSense Depth Camera D435, Intel, Santa Clara, CA) using dedicated software PeacsCamera version 0.0.2.6769 (Peacs Investments BV, Nieuwerbrug aan den Rijn, The Netherlands).

BSPM was continuously recorded at a 2048-Hz sample frequency. A baseline BSPM was recorded for ∼15 minutes, where the first 4–5 minutes were recorded in the supine position, during which the subject was instructed to perform deep inspiration and expiration hold for 10 seconds. This was followed by the reclined position of around 45° for 4–5 minutes. Hereafter, the subject was instructed to perform 30 squats to increase the heart rate with the electrodes still attached. Immediately after the squats, the subject was positioned in the supine position again and BSPM was recorded for another 4–5 minutes. After ∼1 week, a follow-up BSPM was recorded for 4–5 minutes in the supine position. It was assumed that the depolarization and repolarization sequence in this healthy population would be mostly unchanged within 1 week, while this was enough time for the researchers to not precisely recall the position of the electrodes. The researchers tried to place the electrodes at the same location as during the first BSPM using the 3D camera photographs as a reference. Each BSPM was performed by 1 researcher who was consistently present for all measurements, and in addition, 1 of 2 alternating researchers was present.

### Data processing

**Figure 2:**
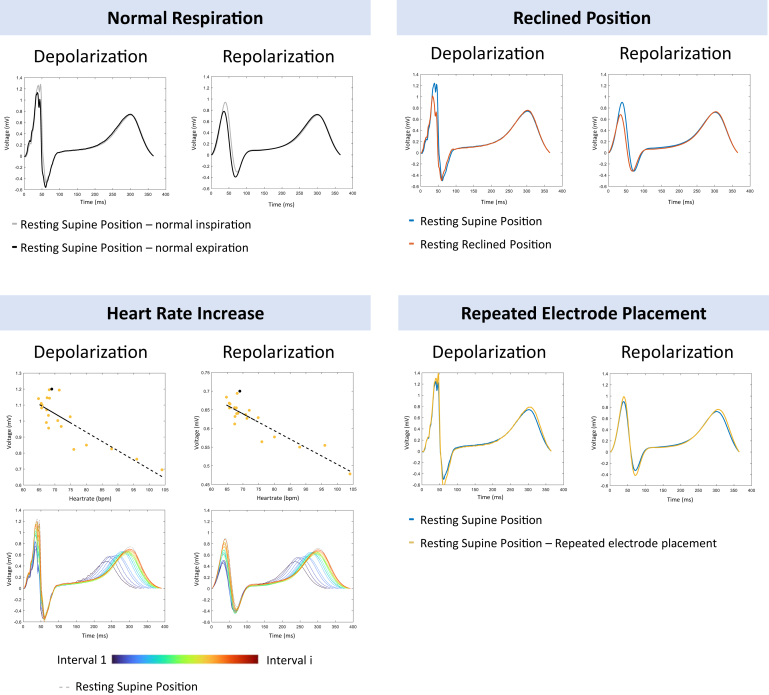
Data analysis methods per condition for depolarization and repolarization analyses. Data analysis of the effect of normal respiration (*upper left panel*), reclined position (*upper right panel*), increased heart rate (*lower left panel*), and repeated electrode placement (lower right panel) is presented. Examples of median beats during inspiration (*gray*), expiration (*dashed line*), reclined position (*orange*), supine position (blue), and repeated electrode placement (*yellow*) are shown. The regression models for depolarization and repolarization are shown in the *upper row* of the *left lower panel*. The *yellow dots* represent the R-, S-, and T-wave amplitudes (in millivolts) derived from the signals in the *bottom row*, and the *black dot* represents the median beat during the supine position. Median beats are shown for the different heart rate intervals during the first 15 seconds (*dark blue*) directly after exercise until the last 15-second interval (*red*). The signals visualized are from lead 45 of subject 4.

BSPM signals were analyzed using MATLAB version R2023B (MathWorks, Natick, MA). The signals were downsampled from 2048 to 1000 Hz using the *resample* function in MATLAB and separately filtered using a Butterworth filter for depolarization (high-pass: cut-off frequency = 0.1 Hz) analysis and repolarization (high-pass: cut-off frequency = 0.1 Hz; low-pass: cut-off frequency = 20 Hz) analysis. The signals were separately filtered to reduce any influence of signal noise in the ST segment while avoiding reduction in amplitude in the QRS segment, as shown in Figure 2.

During the supine position, reclined position, and repeated electrode placement, a median beat was computed for every lead using all beats from 15 seconds of recording. A second median beat was computed during the supine position to determine the validity of our method and the maximum expected differences solely as result of median beat computation. For the exercise-increased heart rate, consecutive median beats were computed over 15-second intervals during the 4- to 5-minute postexercise recording. The heart rate was also determined over each 15-second interval. For the normal respiration condition, 2 median beats were created to represent inspiration and expiration. Individual beats were selected from a 50-second interval during the resting supine position where the 10 beats with the greatest R-wave amplitude were detected and selected to create a median beat for inspiration and the 10 beats with the lowest R-wave amplitude were selected and used to create a median beat for expiration. Normal inspiration and expiration were identified on the basis of observed changes in R-wave amplitude during deep inspiration and expiration hold.

Median beats were created by aligning individual complexes on the R-wave peak as detected using the Pan-Tompkins algorithm.^9^ The root mean square signal, calculated with all 67 median beats, was used to manually annotate the global onset of depolarization (QRS-onset) and end of depolarization (QRS-end). End of repolarization (T-end) was detected using an integration operation method.^10^ All individual beats were baseline corrected between QRS-onset and T-end using linear interpolation before calculating the median. All annotations and median beats were checked manually.

### Data analysis

A summary of all data analysis methods per condition is given in Figure 2. The 2 median beats during the supine position were compared for validation. Normal respiration was investigated by comparing the inspiration median beat with the expiration median beat. The median beats during the reclined position and repeated electrode placement were compared with a median beat during the supine position. For the exercise-increased heart rate condition, all median beats during the 5 minutes postexercise were compared.

The median beats were compared by determining the differences in the amplitudes of the R-, S-, and T-waves across all individual leads. R- and S-wave peaks were automatically detected and considered valid if they met criteria as previously established.^11^ If an R-/S-wave peak did not meet these criteria, the amplitude was set to 0. All annotations were checked manually and corrected if necessary, based on mutual agreement of the researchers. T-wave peaks were detected as the maximum or minimum deflection between QRS-end +70 ms until T-end.

To assess the effect of an increased heart rate, the association between the heart rate and the R-, S-, and T-wave amplitudes was modeled per lead by using a simple linear regression model. The regression coefficients were calculated for each lead and R-, S-, and T-wave peaks separately, and they were expressed in millivolts per beat per minute. An amplitude that became more positive or less negative with an increasing heart rate resulted in a positive regression coefficient. An amplitude that became less positive or more negative with an increasing heart rate resulted in a negative regression coefficient (Figure 2).

A median heatmap of all subjects, males, and females was created for each condition to demonstrate the areas where the median peak amplitude across subjects became more positive or less negative (red) or less positive or more negative (blue) or where the regression coefficient was most positive (red) or most negative (blue). Box plots were used to display the variation in values across each lead. Also, the maximum differences in R-, S-, and T-wave amplitudes between the different conditions were determined for each subject. Values are presented as median (interquartile range [IQR]) and were stratified by sex.

## Results

### Baseline characteristics

In total, 10 study subjects were included (5 (50.0%) male; median age 26 years [IQR 28–30 years]). Subjects had a median heart rate at rest of 61 beats/min (IQR 54–65 beats/min), which increased to 107 beats/min (IQR 97–108 beats/min) after the squats. During follow-up BSPM, the median heart rate was 62 beats/min (IQR 57–66 beats/min). PQ interval, QRS duration, corrected QT duration, and QRS axis were within the normal range for all subjects. The baseline characteristics of the study population are summarized in Table 1.

**Table 1:** Baseline characteristics of all subjects and stratified by sex

Characteristic	All (n=10)	Male (n=5)	Female (n=5)
Age (y)	28 (26, 30)	30 (29, 31)	26 (26, 27)
Body mass index (kg/m^2^)	22 (21, 23)	22 (21, 23)	22 (19, 23)
Time between the first and the second measurement (d)	7 (7, 9)	8 (7, 9)	7 (7, 7)
BSPM characteristics at baseline			
PR interval (s)	0.16 (0.14, 0.17)	0.17 (0.15, 0.20)	0.14 (0.14, 0.16)
QRS duration (s)	0.10 (0.09, 0.10)	0.10 (0.09, 0.11)	0.10 (0.09, 0.10)
QTc duration (s)	0.40 (0.37, 0.41)	0.40 (0.40, 0.41)	0.37 (0.37, 0.39)
QRS axis (deg)	72 (61, 80)	70 (55, 79)	75 (65, 80)
Heart rate			
*BSPM 1*			
Baseline (beats/min)	61 (54, 65)	65 (64, 67)	55 (54, 57)
Exercise (beats/min)	107 (97, 108)	108 (104, 113)	106 (80, 107)
*BSPM 2*			
Baseline (beats/min)	62 (57, 66)	65 (64, 66)	57 (56, 58)

Values are presented as median (IQR).

BSPM = body surface potential mapping; QTc = corrected QT.

Figure 3, Figure 4, Figure 5 show the effects of all conditions on R-, S-, and T-wave amplitudes in 64 leads for all subjects and stratified by sex. Figure 6 shows the effects of an increased heart rate on the R-, S-, and T-wave amplitudes. Online Supplemental Appendix 1, Figures 1–15 show the median and IQR values in each lead for each condition.

**Figure 3:**
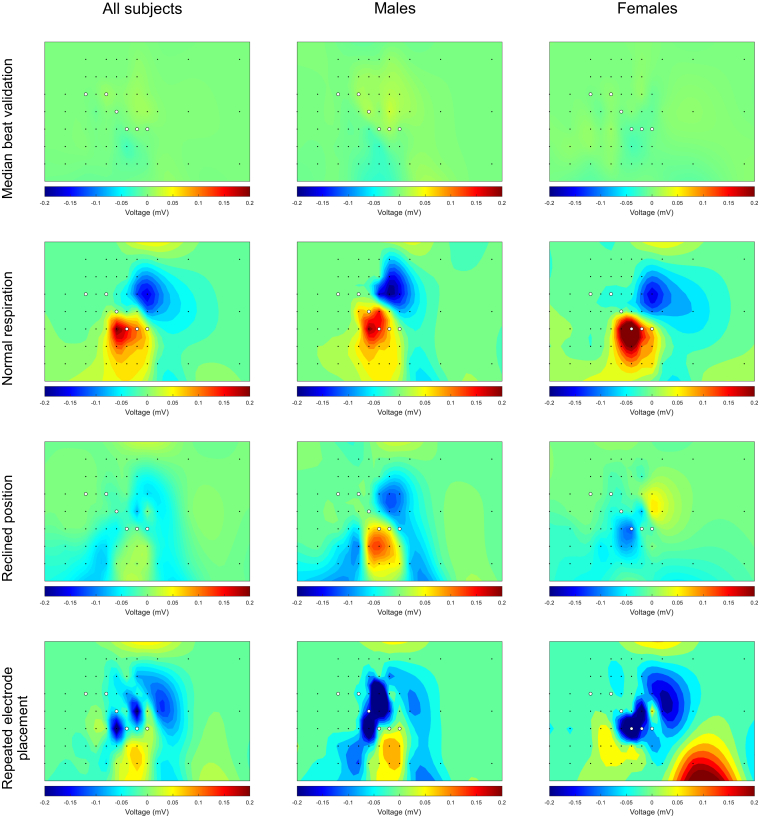
Heatmaps of the signal variation in R-wave amplitude between the 2 median beats during the supine position (*first row*), normal respiration (*second row*), reclined position (*third* row), and repeated electrode placement (*third row*) for all subjects (*left column*), males (*middle column*), and females (*right column*). The *black dots* represent the 64 torso electrodes (see Figure 1), and the *white dots* represent the standard positions of precordial leads V_1_–V_6_. *Blue* represents an R-wave amplitude decrease and *red* an increase.

**Figure 4:**
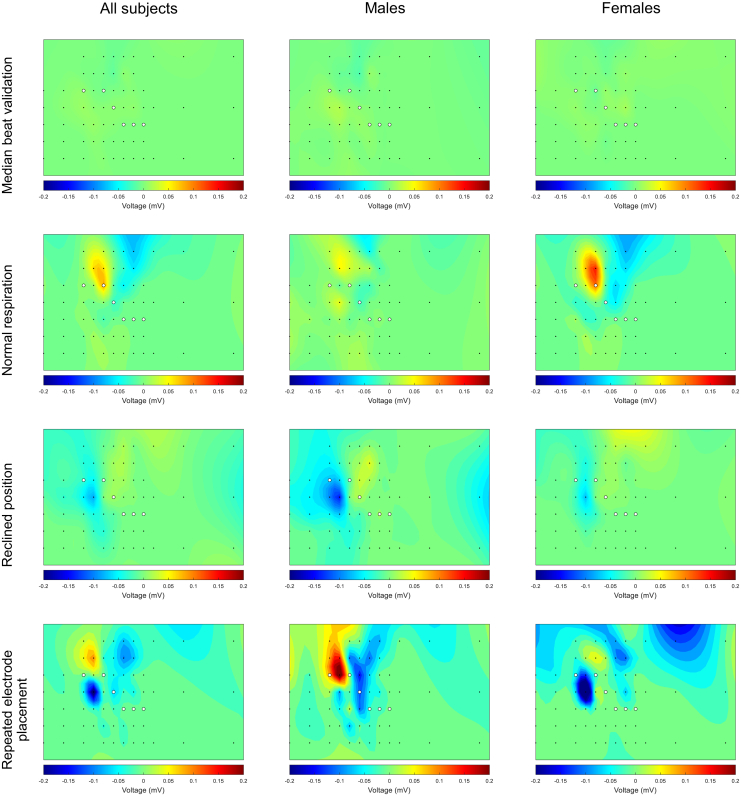
Heatmaps of the signal variation in S-wave amplitude between the 2 median beats during the supine position (*first row*), normal respiration (*second row*), reclined position (*third row*), and repeated electrode placement (*third row*) for all subjects (*left column*), males (*middle column*), and females (*right column*). The *black dots* represent the 64 torso electrodes (see Figure 1), and the *white dots* represent the standard positions of precordial leads V_1_–V_6_. *Blue* represents an S-wave amplitude that became more negative and *red* an S-wave amplitude that became less negative.

**Figure 5:**
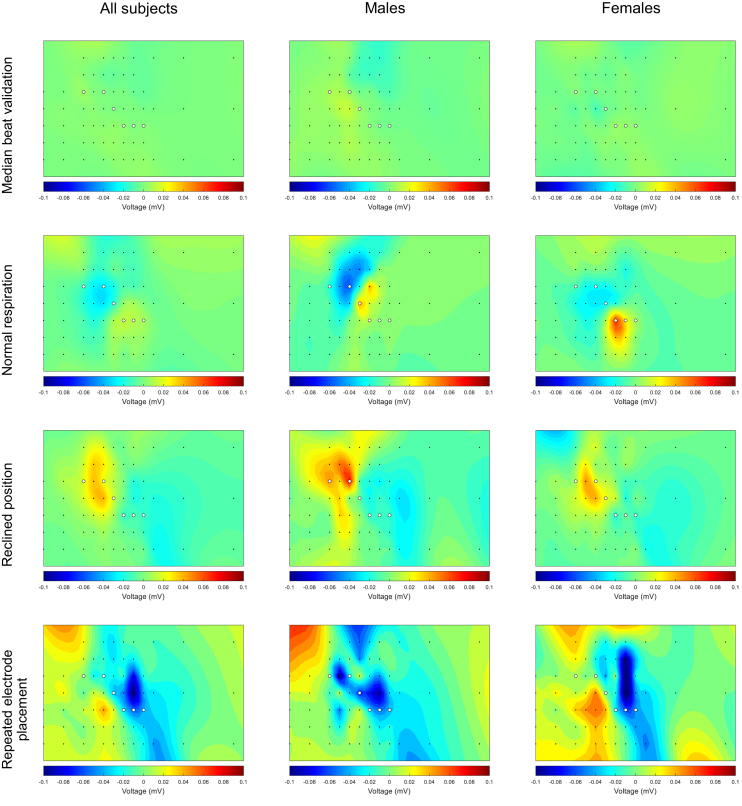
Heatmaps of the signal variation in T-wave amplitude between the 2 median beats during the supine position (*first row*), normal respiration (*second row*), reclined position (third row), and repeated electrode placement (*third row*) for all subjects (*left column*), males (*middle column*), and females (*right column*). The *black dots* represent the 64 torso electrodes (see Figure 1), and the *white dots* represent the standard positions of precordial leads V_1_–V_6_. *Blue* represents an T-wave amplitude that became less positive or more negative and *red* a T-wave amplitude that became more positive or less negative.

**Figure 6:**
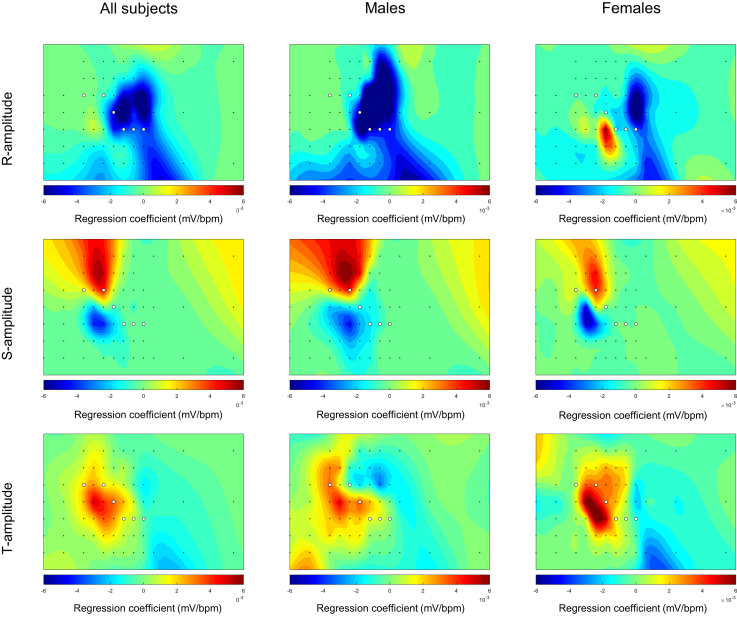
Heatmaps of the regression coefficients for the change in the amplitude of the R wave (*first row*), S wave (*second row*), and T wave (*third row*) due to an increased heart rate for all subjects (*left column*) and specifically for males (*middle column*) and females (*right column*). The *black dots* represent the 64 torso electrodes (see Figure 1), and the *white dots* represent the standard positions of precordial leads V_1_–V_6_. The heatmaps show a negative (*blue*) or positive (*red*) relation (regression coefficient) between amplitude and an increased heart rate.

### Median beat validation

The values of the maximum increases and decreases in R-, S-, and T-wave amplitudes between the 2 median beats during resting supine position are provided in Table 2. The greatest median increase in R-wave amplitude was detected in lead 52 (lead strip V6, second row) in all subjects, lead 47 (lead strip V5, fourth row) in males, and lead 27 (lead strip V2, fifth row) in females. The greatest decrease was detected in lead 42 (lead strip V4, sixth row) in all subjects, males, and females.

**Table 2:** Maximum increase or decrease in amplitude for median beat validation

Maximum increase or decrease	All (n=10)	Males (n=5)	Females (n=5)
R-wave amplitude			
Increase	0.04 (0.02, 0.05)	0.05 (0.02, 0.11)	0.03 (0.03, 0.05)
Decrease	−0.05 (−0.06, −0.02)	−0.05 (−0.05, −0.04)	−0.03 (−0.09, −0.02)
S-wave amplitude			
Increase	0.04 (0.02, 0.07)	0.04 (0.02, 0.07)	0.03 (0.03, 0.07)
Decrease	−0.04 (−0.06, −0.03)	−0.06 (−0.07, −0.03)	−0.04 (−0.05, −0.02)
T-wave amplitude			
Increase	0.01 (0.01, 0.03)	0.03 (0.01, 0.03)	0.01 (0.01, 0.02)
Decrease	−0.02 (−0.02, −0.01)	−0.02 (−0.06, −0.01)	−0.02 (−0.02, −0.01)

The increase or decrease represents the maximum difference in R-, S-, and T-wave amplitudes in all leads for each subject in millivolts. For each subject, this maximum can occur in a different lead. The final value reported in the table is the median (IQR) of these maximum changes across all subjects.

The greatest increase in S-wave amplitude was observed in lead 14 (lead strip V1, fourth row) in all subjects, lead 20 (sternum strip, third row) in males, and lead 47 (lead strip V5, fourth row) in females. The greatest decrease was detected in lead 31 (lead strip V3, second row) in all subjects and in males; in females, it was observed in lead 30 (lead strip V3, first row).

The greatest increase in T-wave amplitude was observed in lead 13 (lead strip V1, third row) in all subjects, lead 26 (lead strip V2, fourth row) in males, and lead 41 (lead strip V4, fifth row) in females. The greatest decrease was observed in lead 24 (lead strip V2, second row) in all subjects, lead 44 (lead strip V5, first row) in males, and in lead 26 (lead strip V2, fourth row) in females.

### Normal respiration

The values of the maximum increases and decreases in R-, S-, and T-wave amplitudes due to normal respiration are provided in Table 3. The largest median increase in R-wave amplitude was observed in lead 34 (lead strip V3, fifth row) in all subjects and in lead 41 (lead strip V4, fifth row) in both males and females. The greatest decrease was observed in lead 51 (lead strip V6, first row) in the whole group and in females; in males, the greatest decrease in R-wave amplitude was observed in lead 46 (lead strip V5, third row).

**Table 3:** Maximum increase or decrease in amplitude for normal respiration

Maximum increase or decrease	All (n=10)	Males (n=5)	Females (n=5)
R-wave amplitude			
Increase	0.32 (0.08, 0.55)	0.25 (0.11, 0.63)	0.39 (0.08, 0.45)
Decrease	−0.20 (−0.25, −0.12)	−0.25 (−0.37, −0.13)	−0.18 (−0.22, −0.09)
S-wave amplitude			
Increase	0.18 (0.10, 0.22)	0.22 (0.09, 0.43)	0.16 (0.12, 0.21)
Decrease	−0.13 (−0.21, −0.09)	−0.21 (−0.25, −0.08)	−0.11 (−0.15, −0.09)
T-wave amplitude			
Increase	0.09 (0.02, 0.14)	0.14 (0.02, 0.15)	0.08 (0.02, 0.09)
Decrease	−0.06 (−0.10, −0.04)	−0.10 (−0.12, −0.07)	−0.04 (−0.06, −0.03)

The increase or decrease represents the maximum difference in R-, S-, and T-wave amplitudes in all leads for each subject in millivolts. For each subject, this maximum can occur in a different lead. The final value reported in the table is the median (IQR) of these maximum changes across all subjects.

The greatest increase in S-wave amplitude was observed in lead 25 (lead strip V2, third row) in all subjects, lead 18 (sternum strip, first row) in males, and lead 24 (lead strip V2, second row) in females. The greatest decrease was observed in lead 44 (lead strip V5, first row) in all subjects and in females; in males, the greatest decrease was observed in lead 37 (lead strip V4, first row).

The greatest increase in T-wave amplitude on a group level and in females was observed in lead 41 (lead strip V4, fourth row); in males, the greatest increase was observed in lead 33 (lead strip V3, fourth row). The greatest decrease was observed in lead 26 (lead strip V2, fourth row) in all subjects and in females; in males, the greatest decrease was observed in lead 25 (lead strip V2, third row).

### Reclined position

The values of the maximum increases and decreases in R-, S-, and T-wave amplitudes due to a reclined position are provided in Table 4. The greatest median increase in R-wave amplitude was observed in lead 49 (lead strip V5, sixth row) in all subjects, lead 42 (lead strip V4, sixth row) in males, and lead 52 (lead strip V6, second row) in females. The greatest decrease was observed in lead 57 (lead strip V5, fourth row) in all subjects, lead 46 (lead strip V5, third row) in males, and lead 41 (lead strip V4, fifth row) in females.

**Table 4:** Maximum increase or decrease in amplitude for reclined position

Maximum increase or decrease	All (n=10)	Males (n=5)	Females (n=5)
R-wave amplitude			
Increase	0.15 (0.08, 0.20)	0.19 (0.14, 0.42)	0.09 (0.08, 0.13)
Decrease	−0.23 (−0.28, −0.15)	−0.26 (−0.38, −0.22)	−0.15 (−0.27, −0.14)
S-wave amplitude			
Increase	0.12 (0.07, 0.18)	0.13 (0.08, 0.19)	0.12 (0.04, 0.14)
Decrease	−0.10 (−0.47, −0.07)	−0.14 (−0.48, −0.07)	−0.10 (−0.22, −0.06)
T-wave amplitude			
Increase	0.06 (0.06, 0.10)	0.10 (0.08, 0.22)	0.06 (0.05, 0.06)
Decrease	−0.05 (−0.06, −0.03)	−0.05 (−0.10, −0.03)	−0.05 (−0.06, −0.03)

The increase or decrease represents the maximum difference in R-, S-, and T-wave amplitudes in all leads for each subject in millivolts. For each subject, this maximum can occur in a different lead. The final value reported in the table is the median (IQR) of these maximum changes across all subjects.

Overall and in females, the greatest S-wave increase was observed in lead 37 (lead strip V4, first row); in males, the greatest increase was observed in lead 38 (lead strip V4, second row). The greatest decrease was observed in lead 20 (sternum strip, third row) in the whole group, males, and females.

The greatest increase in T-wave amplitude was observed in lead 26 (lead strip V2, fourth row) in all subjects, lead 25 (lead strip V2, third row) in males, and lead 19 (sternum strip, second row) in females. The greatest decrease was observed in lead 58 (lead strip Back1, third row) in all subjects, lead 57 (lead strip Back1, second row) in males, and lead 47 (lead strip V5, fourth row) in females.

### Repeated electrode placement

The values of the maximum increases and decreases in R-, S-, and T-wave amplitudes due to repeated electrode placement are provided in Table 5. The greatest median increase in R-wave amplitude was observed in lead 50 (lead strip V5, seventh row) in all subjects, lead 49 (lead strip V5, sixth row) in males, and lead 61 (lead strip Back2, first row) in females. The greatest decrease was observed in lead 34 (lead strip V3, fifth row) in all subjects, lead 39 (lead strip V4, third row) in males, and lead 41 (lead strip V4, fifth row) in females.

**Table 5:** Maximum increase or decrease in amplitude for repeated electrode placement

Maximum increase or decrease	All (n=10)	Males (n=5)	Females (n=5)
R-wave amplitude			
Increase	0.33 (0.18, 0.42)	0.36 (0.20, 0.42)	0.31 (0.18, 0.52)
Decrease	−0.63 (−0.69, −0.22)	−0.63 (−0.71, −0.53)	−0.55 (−0.85, −0.21)
S-wave amplitude			
Increase	0.27 (0.14, 0.46)	0.46 (0.33, 0.52)	0.14 ( 0.13, 0.20)
Decrease	−0.43 (−0.62, −0.25)	−0.53 (−0.62, −0.24)	−0.37 (−0.53, −0.24)
T-wave amplitude			
Increase	0.11 (0.10, 0.17)	0.16 (0.12, 0.17)	0.10 (0.07, 0.14)
Decrease	−0.23 (−0.33, −0.12)	−0.33 (−0.44, −0.24)	−0.12 (−0.16, −0.11)

The increase or decrease represents the maximum difference in R-, S-, and T-wave amplitudes in all leads for each subject in millivolts. For each subject, this maximum can occur in a different lead. The final value reported in the table is the median (IQR) of these maximum changes across all subjects.

The greatest increase in S-wave amplitude was observed in lead 18 (sternum strip, first row) in all subjects, lead 19 (sternum strip, second row) in males, and lead 24 (lead strip V2, second row) in females. Overall and in females, the greatest decrease was observed in lead 20 (sternum strip, third row) and in males in lead 32 (lead strip V3, third row).

On a group level and in females, the greatest increase in T-wave amplitude was observed in lead 27 (lead strip V2, fifth row); in males, the greatest increase was observed in lead 1 (lead strip Vr5, first row). The greatest decrease was observed in lead 47 (lead strip V5, fourth row) on a group level and in females; in males, the greatest decrease was observed in lead 19 (sternum strip, second row).

### Increased heart rate

The values of the maximum positive and maximum negative regression coefficients due to an increased heart rate are provided in Table 6. The greatest median increase in R-wave amplitude was obvserved in lead 21 (sternum strip, fourth row) in all subjects, lead 20 (sternum strip, third row) in males, and lead 34 (lead strip V3, fifth row) in females. The greatest decrease in R-wave amplitude was observed in lead 40 (lead strip V4, fourth row) in all subjects and in males and in lead 52 (lead strip V6, second row) in females.

**Table 6:** Maximum positive or negative regression coefficients for increased heart rate

Maximum positive or negative regression coefficient	All (n=10)	Males (n=5)	Females (n=5)
R-wave amplitude			
Increase	0.002 (0.001, 0.007)	0.001 (4.211e^−4^, 0.002)	0.007 (0.003, 0.009)
Decrease	−0.012 (−0.014, −0.009)	−0.013 (−0.028, −0.011)	−0.009 (−0.013, −0.006)
S-wave amplitude			
Increase	0.007 (0.006, 0.011)	0.006 (0.006, 0.013)	0.008 (0.005, 0.010)
Decrease	−0.006 (−0.007, −0.004)	−0.005 (−0.007, −0.004)	−0.007 (−0.010, −0.004)
T-wave amplitude			
Increase	0.007 (0.004, 0.013)	0.007 (0.004, 0.012)	0.007 (0.006, 0.013)
Decrease	−0.005 (−0.006, −0.003)	−0.006 (−0.009, −0.004)	−0.004 (−0.005, −0.003)

The positive or negative coefficient represents the highest positive or negative regression coefficients for R-, S-, and T-wave amplitudes in all leads for each subject in millivolts/beats/min. For each subject, this maximum can occur in a different lead. The final value reported in the table is the median (IQR) of these maximum changes across all subjects.

The greatest median increase in S-wave amplitude was observed in lead 18 (sternum strip, first row) in all subjects and in lead 24 (lead strip V2, second row) in both males and females. The greatest decrease was observed in lead 27 (lead strip V2, fifth row) in all subjects and in males, while in females, lead 21 (sternum strip, fourth row) showed the greatest decrease.

The greatest increase in T-wave amplitude was observed in lead 20 (sternum strip, third row) in all subjects and in males; in females, the greatest increase in amplitude with an increasing heart rate was observed in lead 27 (lead strip V2, fifth row). The greatest decrease was observed in lead 58 (lead strip Back1, third row) in all subjects and in females; in males specifically, lead 46 (lead strip V5, third row) showed the greatest decrease.

## Discussion

With this study, we were able to demonstrate normal intrapersonal variation between 2 consecutive BSPMs in healthy subjects. To our knowledge, this is the first study to describe the normal inter- and intraindividual variation in BSPM due to a range of factors that are both physiological and external. The results of this study will help to distinguish between subtle ECG signs of disease progression and normal intrapersonal variation when comparing 2 consecutive BSPMs. The greatest variation was observed after repeated electrode placement for both depolarization and repolarization, while a reclined position had the least effect. The effect of an increased heart rate was larger in depolarization than in repolarization.

### Normal respiration

Normal respiration showed a relatively large effect on depolarization (maximum R-wave amplitude increase 0.32 mV and decrease −0.20 mV; maximum S-wave amplitude increase 0.18 mV and decrease −0.13 mV) and a small effect on repolarization (maximum T-wave amplitude increase 0.09 mV, decrease −0.06 mV). Also, the effect on the R-wave amplitude was greater than that of the S-wave amplitude. The effect of normal respiration was small in most leads as previously described by Smit et al,^12^ but a prominent R-wave amplitude increase was visible around precordial leads V_3_–V_6_ and a prominent R-wave amplitude decrease above leads V_3_–V_6_. The local larger changes in amplitude can be explained by the change in distance between electrodes and the heart and by changes in lung conductivity.^5^

A study by Amoore et al^5^ also investigated the effects of respiration in 180-electrode BSPM. They determined the peak maxima and minima during depolarization at inspiration and expiration and observed slightly smaller respiratory-related changes compared with our study. This discrepancy may be attributed to our study population’s relatively young age and low body mass index, which may have contributed to greater amplitude values.

### Reclined position

This study showed some effect of a reclined position on both depolarization (maximum R-wave amplitude increase 0.15 mV and decrease −0.23 mV; maximum S-wave amplitude increase 0.12 mV and decrease −0.10 mV) and repolarization (maximum T-wave amplitude increase 0.06 mV and decrease −0.05 mV), although it was minimal. A study by Bergman et al^13^ described a mean decrease in R-wave amplitude of 0.20 mV in leads V_5_ and V_6_ after a reclined position of 60°.^13^ This is a greater difference than what we observed, and the location of amplitude change is more lateral than we observed. However, we investigated a reclined position of only 45° in this study, which might have reduced the effect and influenced the location of greatest variation as well.

### Repeated electrode placement

Although the electrode placement during follow-up measurement was guided by 3D pictures, there were still substantial differences found in amplitudes between the 2 measurements (maximum R-wave amplitude increase 0.33 mV and decrease −0.63 mV; S-wave amplitude increase 0.27 mV and decrease −0.43 mV; T-wave increase 0.11 mV and decrease −0.23 mV). The greatest variation was observed as a decreased R wave just below standard precordial lead V_3_. The greatest change in T-wave was an amplitude decrease just above precordial lead V_5_. A previous study compared consecutive 12-lead ECGs performed in a routine manner with consecutive ECGs performed with 3D camera–guided live projection to aid in the repositioning of electrodes with the aim to place each electrode at the same place as in the first ECG. Greater differences in waveforms were observed in the routinely recorded ECGs (a correlation coefficient of 0.98 and 0.96 of the QRS complex and STT segment, respectively, during 3D camera–guided ECGs vs correlation coefficients of 0.90 and 0.88 of the QRS complex and the STT segment during routine ECGs). QRS-waveform differences were mostly observed in lead V_3_ in routinely recorded ECGs and in leads V_4_–V_5_ in 3D camera–guided ECGs. For the STT segment, the greatest variations were observed in leads V_4_–V_5_ in routinely recorded ECGs and in lead V_3_–V_4_ in 3D camera-guided ECGs.^14^ The greatest differences we observed were around the same locations but were observed slightly above and below leads V_3_–V_5_. These observations highlight the importance of correct electrode (re)positioning of precordial electrodes, as amplitude variations are greatest in these regions.

### Increased heart rate

With an increased heart rate, both a decrease and an increase in amplitude were found for depolarization (maximum R-wave positive and negative regression coefficients 0.002 and −0.012 mV/beats/min; maximum S-wave positive and negative regression coefficients 0.007 and −0.006 mV/beats/min) and repolarization (maximum T-wave positive and negative regression coefficients 0.007 and −0.005 mV/beats/min), depending on the location of the electrodes. Another study by Miller et al^15^ described QRS potential maps derived from 24-electrode BSPM in 5-ms intervals at rest and during exercise and observed a decrease in amplitude during exercise around the R-wave peak, located at the left inferior position of the body surface. This is in concordance with the amplitude decrease (negative regression coefficient) found in the same region in our study. A decrease in R-wave amplitude in left precordial leads is known as a normal electrocardiographic response to exercise.^16^ Miller et al^15^ also described that the lowest potential value at rest became less negative during exercise at the right superior position of the body surface. This is also in concordance with our study where we observed a less negative amplitude with an increasing heart rate (positive regression coefficient) in the same position of the body surface map.

During repolarization, an increase in T-wave amplitude with an increasing heart rate was found in most leads. The greatest increase in amplitude with an increasing heart rate was observed outside the standard precordial positions, at a mid-sternal location. Miller et al^15^ described different results in BSPM derived T-wave potential maps among subjects; the majority had an increase in T-wave magnitude, while a minority had a decrease or no change in T-wave amplitude during exercise. Overall, as in our study, an amplitude increase with exercise seems more common, although it is important to consider that interpersonal differences may occur (Online Supplemental Appendix 1).

### Sex differences

For depolarization and repolarization, males generally showed greater differences in amplitude than did females. When comparing the heatmap patterns between the 2 sexes for each condition, the patterns are similar, although there are some differences. A component of these differences could be explained by female anatomy, resulting in a more variable torso anatomy and distance of each electrode to the heart while also influencing the position of the electrodes on the torso.

### Interpersonal variation

The interpersonal variation per lead was minimal when comparing the 2 median beats in the resting supine position for R, S, and T waves. A greater interpersonal variation was observed during the reclined position and normal respiration. The most significant dispersion, and thus the greatest interpersonal variation in amplitude changes, was observed during repeated electrode placement. This is likely due to differences in accuracy in electrode placement between the 2 measurements and between subjects, which is also shown in the heatmaps, where the minima and maxima are more randomly distributed and not just located in the electrodes located closest to the heart. Nevertheless, our 3D camera–guided method has probably reduced errors in electrode placement. It is expected that the observed differences would have been greater without the use of 3D pictures.

Across all conditions, the S-wave amplitude tended to have a greater variation in the right-sided leads between subjects while the observed R-wave amplitude changes were more variable in the left-sided leads. This observation aligns with expectations, as S-waves are generally more prominent in leads V_1_–V_2_ while R-waves are more prominent in leads V_5_–V_6_. In contrast, T-wave amplitude changes were more evenly distributed across all leads. In general, the largest changes were most often observed in the middle leads of each strip. These leads have the greatest amplitudes, as they are located closest to the heart.

Some leads consistently showed either an increase or a decrease in amplitude in all subjects, while in other leads, both an increase and a decrease could be observed depending on the subject (as can be seen, for instance, in the R-wave of lead 41 during the reclined position) (Online Supplemental Appendix 1, Figure 7). Therefore, rather than solely focusing on whether an individual shows an increase or decrease in amplitude between measurements, it is important to consider if these observed changes are within the expected range of interpersonal variation or outside this range.

### Methodological considerations

Our median beat computation method demonstrated high precision, as shown by the small differences in R-, S-, and T-wave amplitudes (Table 2) between the 2 median beats during the resting supine position. The differences identified between the supine position and other conditions can thus be attributed to the conditions themselves rather than to the inaccuracy of our methodology. This high precision emphasizes the importance of computing a median beat to eliminate the effects of respiration instead of analyzing individual beats, as this leads to a possibility of selecting a beat during inspiration and a beat during expiration. As observed in our findings during normal respiration, this can result in important differences.

### Limitations

In this study, we detected the R-/S-wave peaks in all individual leads for each subject. A drawback of this method is the interpersonal variation in the presence of R- or S-waves per lead (Online Supplemental Appendix 1). By comparing only the peaks, other morphological changes that could have been present, for example, an Rʹ-wave, may have gone undetected. Determining differences across the whole QRS complex or the whole STT segment might have discovered more changes and variation in BSPM signals. However, the R-, S-, T-wave amplitudes are more clinically used parameters and are therefore easier to translate to clinical practice.

Furthermore, we used a different analysis method for the increased heart rate, as we did not determine the difference between 2 median beats (in millivolts) but determined a trend across a spectrum of different heart rates (in millivolts per beats per minute). This makes it slightly more difficult to compare with other conditions. However, the current method provided the possibility to evaluate the full effect of heart rate increase regardless of the differences between baseline and maximum heart rates between subjects.

Lastly, limitations of this study are the small sample size and the relatively young age of the study population. A larger cohort with a wider age range could have provided more accurate information. Nevertheless, we have an equal representation of male and female study participants, making the results of this study applicable to both sexes.

### Future perspective

With this study we were able to demonstrate for the first time the limits of normal intrapersonal variation in 67-lead BSPM. One of the current limitations of BSPM in clinical practice is that a normal reference is not well described; thus, the results of this study will be useful when comparing 2 BSPMs over time. Any exceeding of these determined limits might be due to the progression of cardiac disease instead of normal variation.

## Conclusion

This study showed the effects of normal respiration, a reclined position, an increased heart rate, and repeated electrode placement on the amplitudes of the body surface potentials. The normal intrapersonal variation in QRS and T-wave amplitudes for all 67 leads under these conditions was established. The greatest variation in amplitude was observed during normal respiration and repeated electrode placement. An increase in heart rate can result in both an increase and a decrease in QRS and T-wave amplitudes, depending on the location of the electrode.

## Appendix Supplementary Data

Supplemental Figure1-15
