# Supplementary material for: Intra- and interpersonal variation in body surface potentials of healthy subjects
Source: Heart Rhythm O2. 2025 Jan 31;6(4):450–62. doi: 10.1016/j.hroo.2025.01.016 (PMC12047564; doi:10.1016/j.hroo.2025.01.016)
Supplement: Supplemental Figure1-15 [file mmc1.docx]

**Appendix 1**


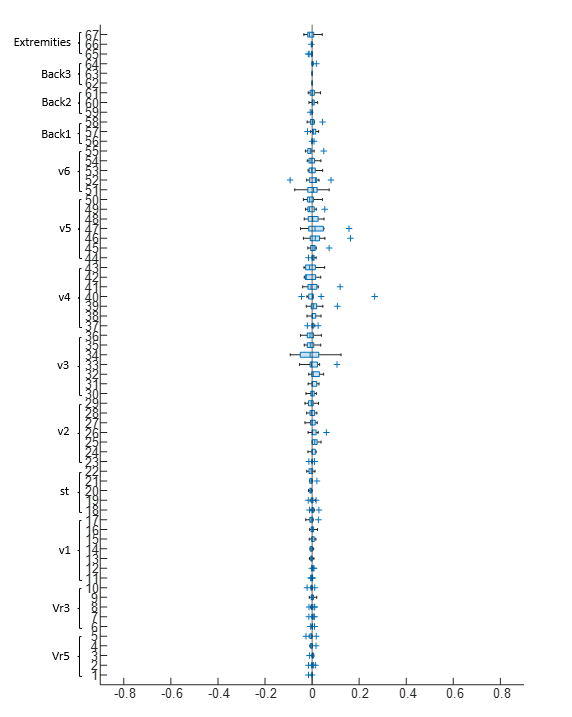


**Supplemental Figure 1.** Boxplots of differences in R-amplitude in mV for median beat validation for all 67 leads.


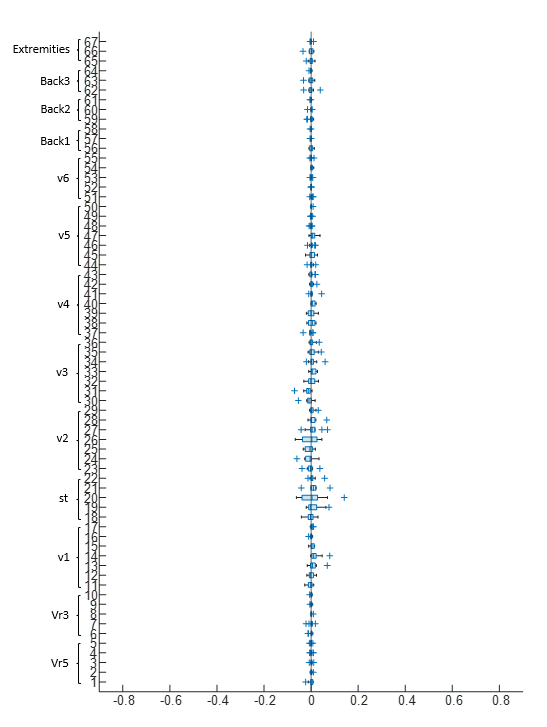


**Supplemental Figure 2.** Boxplots of differences in S-amplitude in mV for median beat validation for all 67 leads.

**
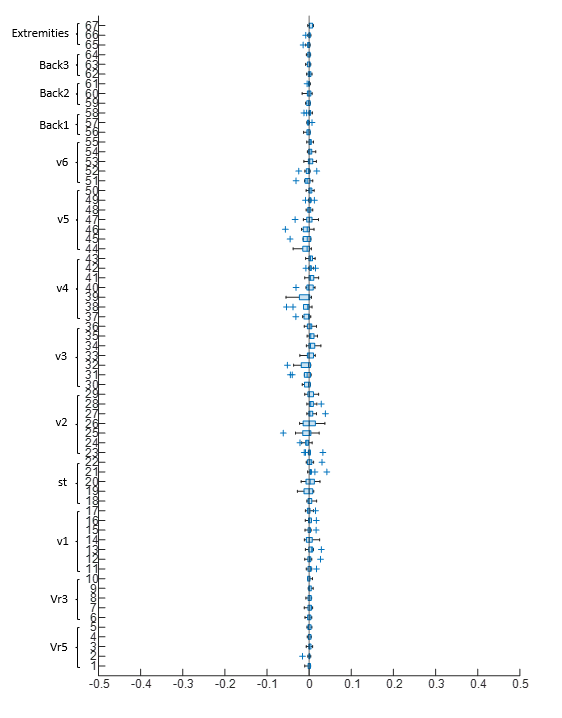
**

**Supplemental Figure 3.** Boxplots for differences in T-amplitude in mV for median beat validation for all 67 leads.

**
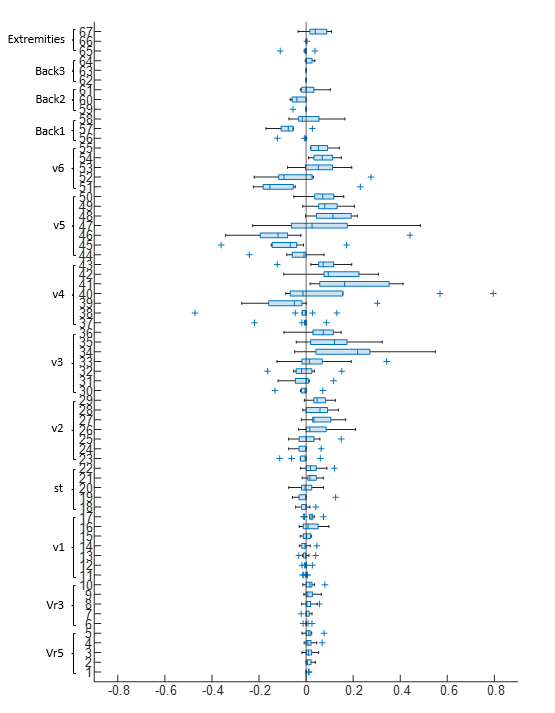
**

**Supplemental Figure 4.** Boxplots of differences in R-amplitude in mV for normal respiration for all 67 leads.


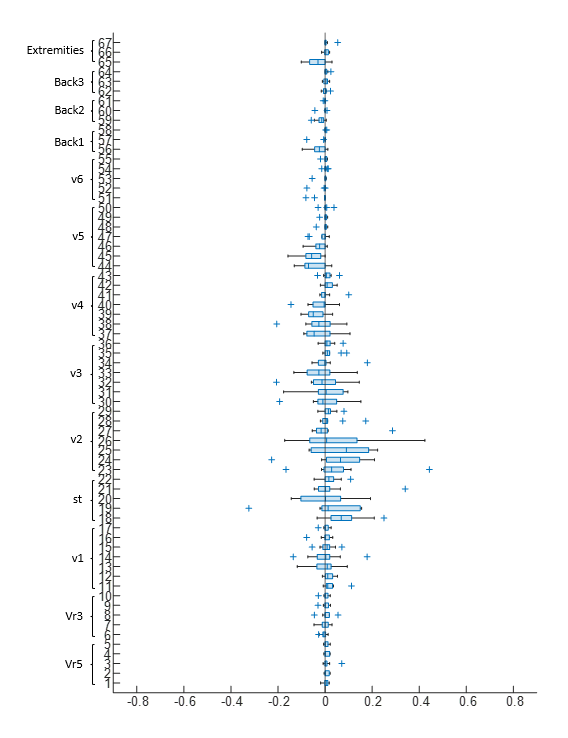


**Supplemental Figure 5.** Boxplots of differences in S-amplitude in mV for normal respiration for all 67 leads.

**
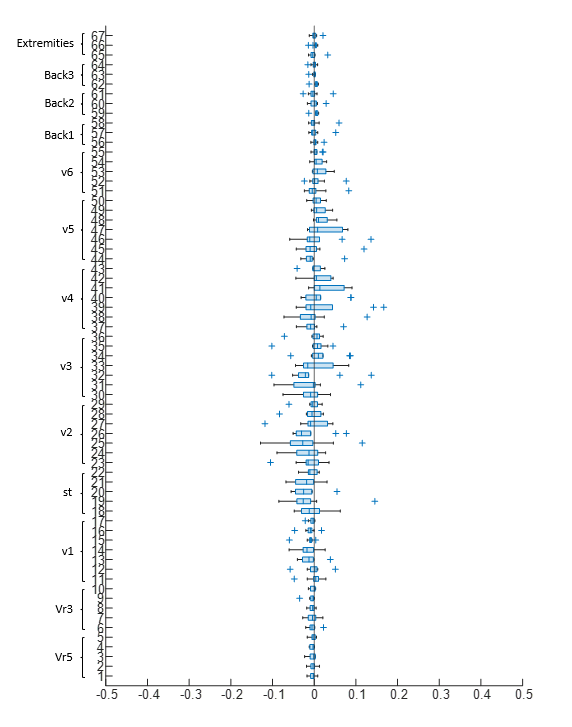
**

**Supplemental Figure 6.** Boxplots of differences in T-amplitude in mV for normal respiration for all 67 leads.


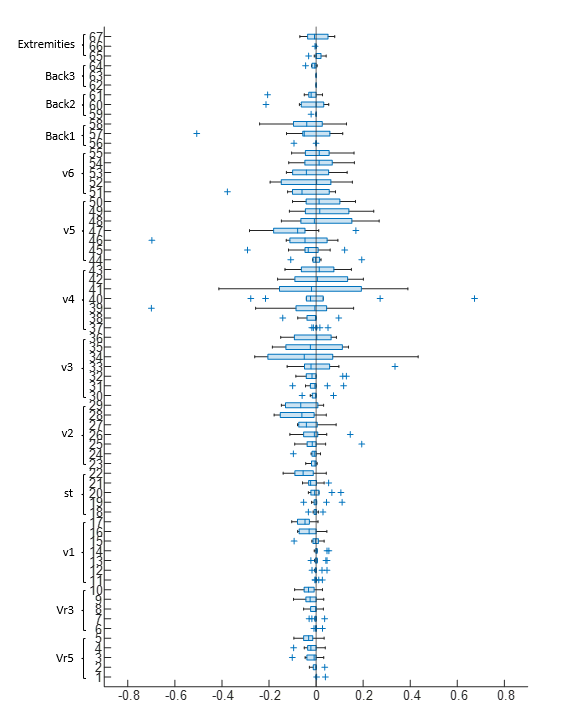


**Supplemental Figure 7.** Boxplots of differences in R-amplitude in mV for reclined position for all 67 leads.


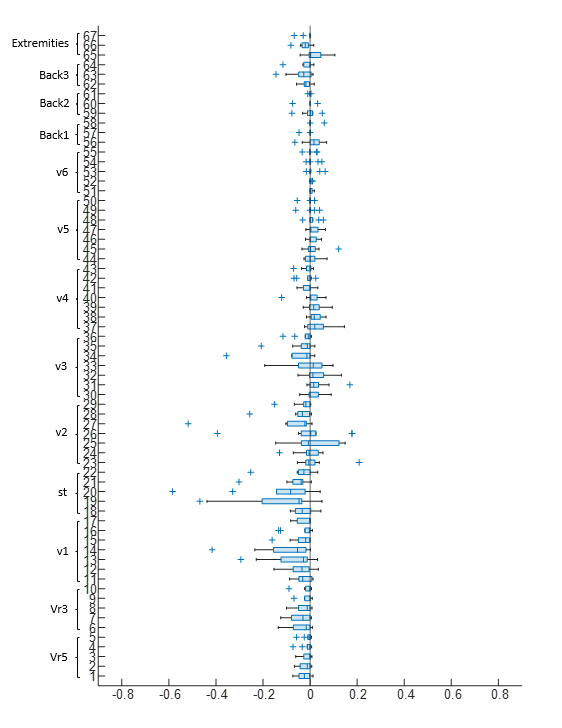


**Supplemental Figure 8.** Boxplots of differences in S-amplitude in mV for reclined position for all 67 leads.

**
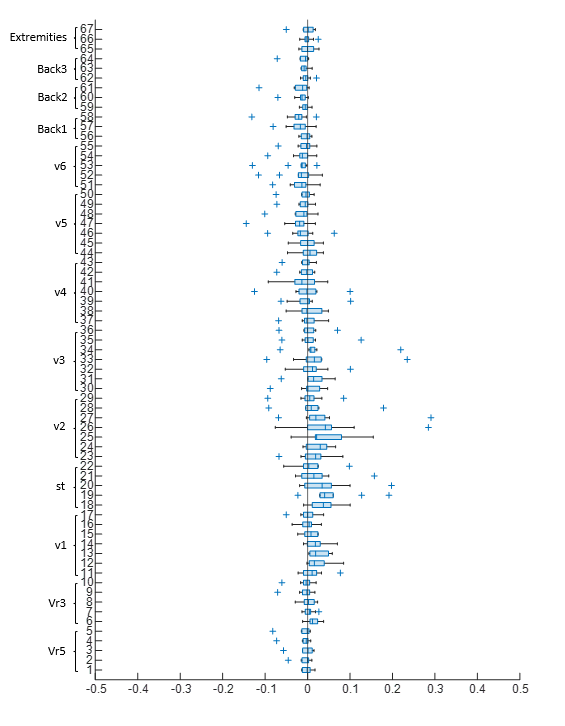
**

**Supplemental Figure 9.** Boxplots of differences in T-amplitude in mV for reclined position for all 67 leads.


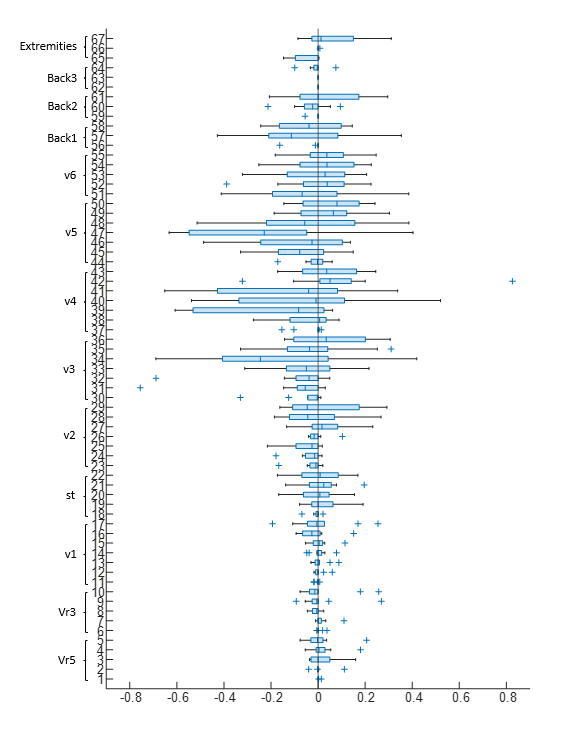


**Supplemental Figure 10.** Boxplots of differences in R-amplitude in mV for repeated electrode placement for all 67 leads.


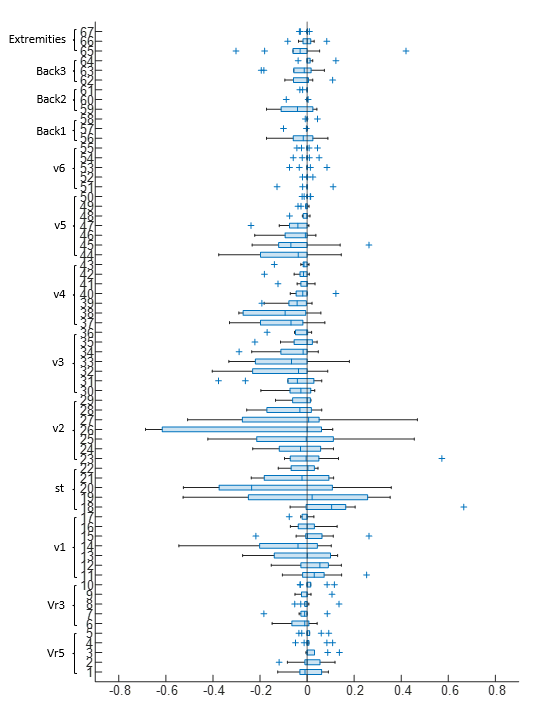


**Supplemental Figure 11.** Boxplots of differences in S-amplitude in mV for repeated electrode placement for all 67 leads.

**
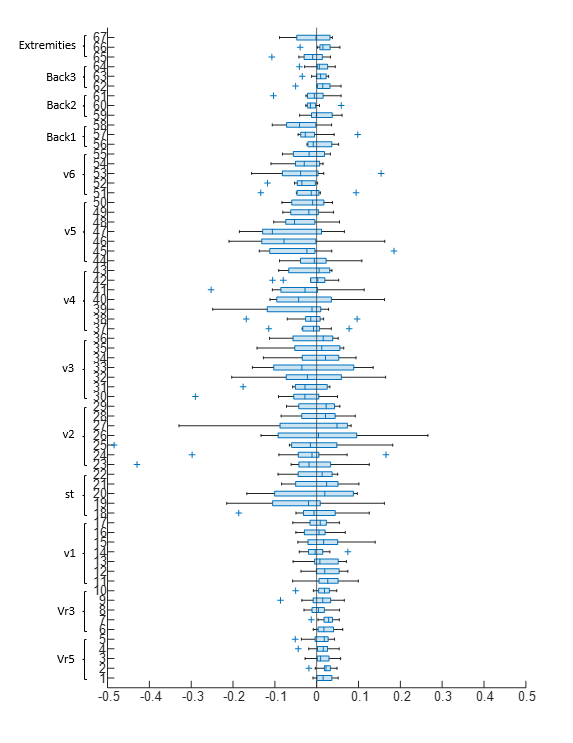
**

**Supplemental Figure 12.** Boxplots of differences in T-amplitude in mV for repeated electrode placement for all 67 leads.


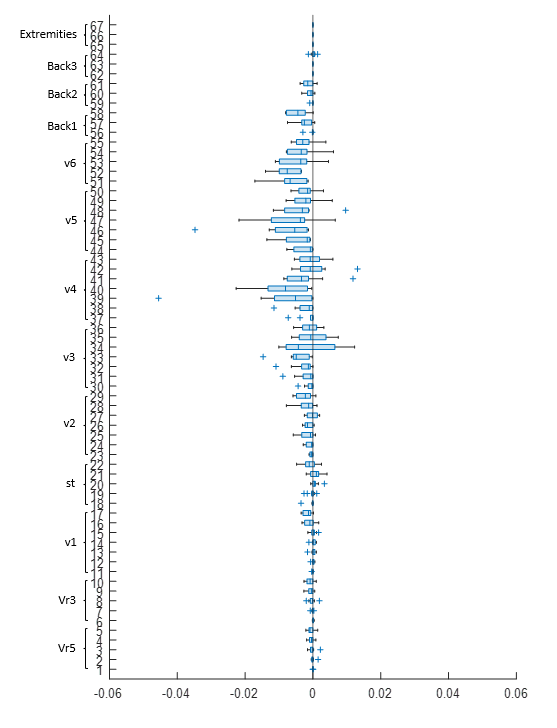


**Supplemental Figure 13.** Boxplots of the regression coefficients (mV/bpm) for the change in R-wave amplitude due to an increased heart rate for all 67 leads.


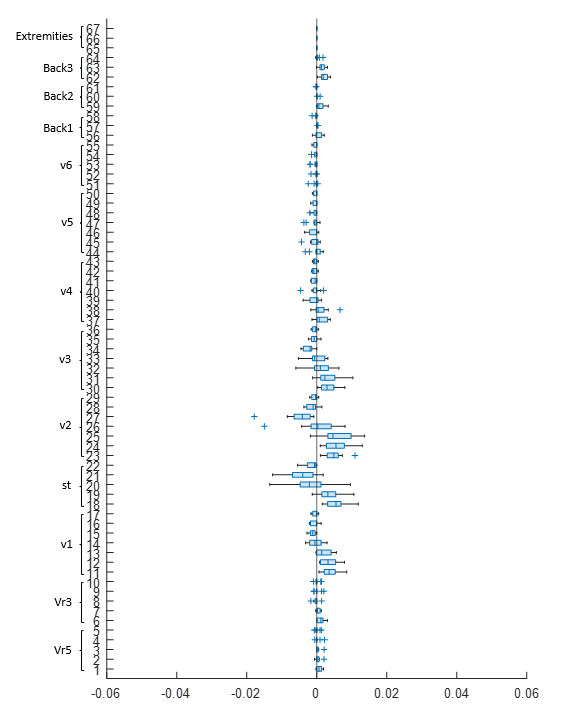


**Supplemental Figure 14.** Boxplots of the regression coefficients (mV/bpm) for the change in S-wave amplitude due to an increased heart rate for all 67 leads.


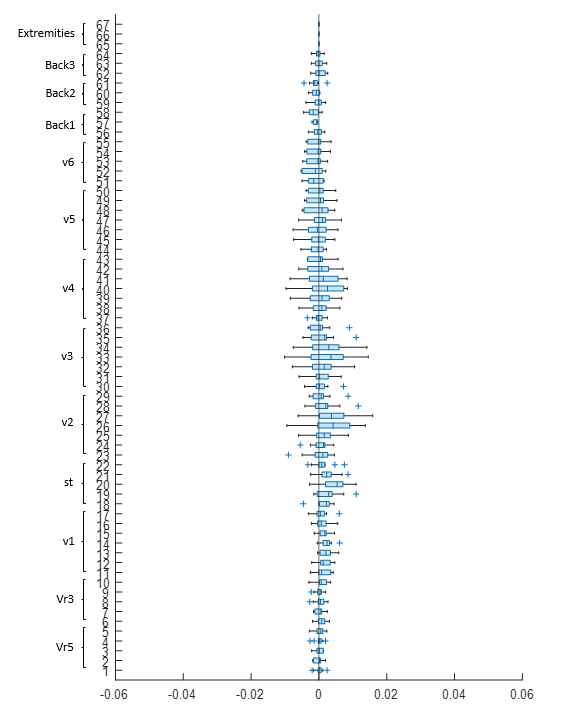


**Supplemental Figure 15.** Boxplots of the regression coefficients (mV/bpm) for the change in T-wave amplitude due to an increased heart rate for all 67 leads.
